# Characterization of claustral neurons by comparative gene expression profiling and dye-injection analyses

**DOI:** 10.3389/fnsys.2014.00098

**Published:** 2014-05-23

**Authors:** Akiya Watakabe, Sonoko Ohsawa, Noritaka Ichinohe, Kathleen S. Rockland, Tetsuo Yamamori

**Affiliations:** ^1^Division of Brain Biology, National Institute for Basic BiologyOkazaki, Japan; ^2^Department of Basic Biology, The Graduate University for Advanced Studies (Sokendai)Hayama, Japan; ^3^Department of Ultrastructural Research, National Center of Neurology and Psychiatry, National Institute of NeuroscienceKodaira, Japan; ^4^Department of Anatomy and Neurobiology, Boston University School of MedicineBoston, MA, USA

**Keywords:** non-human-primate, layer 6, neocortex, migration, ctgf, Gng2, Nr4a2

## Abstract

The identity of the claustrum as a part of cerebral cortex, and in particular of the adjacent insular cortex, has been investigated by connectivity features and patterns of gene expression. In the present paper, we mapped the cortical and claustral expression of several cortical genes in rodent and macaque monkey brains (*nurr1, latexin, cux2*, and *netrinG2*) to further assess shared features between cortex and claustrum. In mice, these genes were densely expressed in the claustrum, but very sparsely in the cortex and not present in the striatum. To test whether the cortical vs. claustral cell types can be distinguished by co-expression of these genes, we performed a panel of double ISH in mouse and macaque brain. *NetrinG2* and *nurr1* genes were co-expressed across entire cortex and claustrum, but *cux2* and *nurr1* were co-expressed only in the insular cortex and claustrum. *Latexin* was expressed, in the macaque, only in the claustrum. The *nurr1*^+^ claustral neurons expressed *VGluT1*, a marker for cortical glutamatergic cells and send cortical projections. Taken together, our data suggest a partial commonality between claustral neurons and a subtype of cortical neurons in the monkey brain. Moreover, in the embryonic (E110) macaque brain, many *nurr1*^+^ neurons were scattered in the white matter between the claustrum and the insular cortex, possibly representing their migratory history. In a second set of experiments, we injected Lucifer Yellow intracellularly in mouse and rat slices to investigate whether dendrites of insular and claustral neurons can cross the border of the two brain regions. Dendrites of claustral neurons did not invade the overlying insular territory. In summary, gene expression profile of the claustrum is similar to that of the neocortex, in both rodent and macaque brains, but with modifications in density of expression and cellular co-localization of specific genes.

## Introduction

Historically, there have been long debates about the identity of the claustrum as belonging with cortex or basal ganglia (see Edelstein and Denaro, 2004; Miyashita et al., 2005; Pirone et al., 2012). Classic morphological studies, for example, have shown that the most common cell type in the claustrum is a type I cell with spiny dendrites and with axon projecting at a distance, as well as with local collaterals (summarized in Crick and Koch, 2005). Owing to rapid advances in molecular data, we now have several molecular markers that can help to clarify the developmental and evolutionary identity of the main claustral neuron subtypes. For example, Mathur and coworkers searched for claustral molecular markers by proteomic approach (Mathur et al., 2009). Of particular note, in this paper, are two genes, latexin, a carboxypeptidase inhibitor (Arimatsu et al., 1992), and nurr1, a nuclear receptor subtype of transcription factor. Both these genes exhibit a strikingly similar expression pattern in the rodent brain; namely, continuous with dense expression in the claustrum and endopiriform nucleus, they are expressed in layer 6 of lateral neocortex (e.g., S2). Despite sparse expression in the neocortex, these two genes are co-expressed in the same cells (nurr 1 has additional expression in layer 6b), and these cells are associated with cortical projections (Arimatsu et al., 2003; Bai et al., 2004; Watakabe et al., 2007). Given the shared features of gene expression and extrinsic connectivity, the possibility arises that Latexin^+^/nurr1^+^ neurons in the cortical deep layers and the claustrum may be categorized as the same subclass of neurons.

To examine the evolutionarily conserved expression of these genes, we previously performed ISH analyses in the macaque brain (Watakabe et al., 2007; Watakabe, 2009). By this search, we found that nurr1^+^ neurons are present in both the claustrum and cortex in the macaque brain, like in the rodent. However, as opposed to lateral-restricted expression in the rodent brain, nurr1 mRNA was expressed in layer 6 across the entire neocortex in macaques, even while latexin mRNA was not detected in the neocortical regions in this species. The differential gene expression in rodent and macaque led us to further investigations to clarify the evolutionary fingerprint of the claustral neurons.

In this paper, we addressed the question of whether the claustrum is more appropriately viewed as cortical or basal ganglial by two approaches. First, we selected additional molecular markers that are enriched in the claustrum in either rodents or macaques with the intention of extending the comparison of cortical and claustral gene expression phenotypes in these two species: Cux2 is a transcription factor that specifies upper layer neuron fate (Nieto et al., 2004; Cubelos et al., 2010; Franco et al., 2012). It is, however, also expressed in the deep layers of the lateral cortex of rodents, where it exhibits a “latexin-like” expression pattern. NetrinG2 is an axon guidance molecule implicated in synapse specification (Nishimura-Akiyoshi et al., 2007). Netrin G2 mRNA in monkeys is expressed in layer 6 of the insular cortex and claustrum (Miyashita et al., 2005).

We aimed to clarify by double ISH whether these genes exhibit similar distribution patterns as that of nurr1 in the two species. Whether they are co-expressed outside claustrum would be a good measure to understand the significance of their expression in both cortex and claustrum. In addition, we reexamined the latexin mRNA expression in the insular/claustral regions of the macaque brain. We also examined the expression of the nurr1 gene in the embryonic monkey and postnatal mouse cortex to see if we could differentiate cortical and claustral neurons in the earlier developmental time point.

As a second approach, we examined the morphology of claustral neurons to ascertain whether their dendrites extend beyond the insular/claustral border in rodents. If the claustral neurons exhibit long dendrites that cross into insular cortex, this could be taken as strong support for a cortical identity.

## Materials and methods

Tracer injection into the adult macaque monkey was performed in RIKEN Institute in accordance with the protocol approved by the Experimental Animal Committee of the RIKEN Institute, which was also concordant with the National Institutes of Health Guide for the Care and Use of Laboratory Animals (NIH Publications No. 80-23), revised 1996. Adult macaque monkey brain sections for ISH were obtained from the samples that were used in a previous study (Watakabe et al., 2007). Embryonic macaque monkey brain was obtained from Tsukuba Primate Research Center, National Institute of Infectious Diseases. Tracer injection into rat brain and perfusion of mice and rats for ISH and Lucifer Yellow injection followed the animal care guidelines of the National Institute for Basic Biology and National Institute for Physiological Sciences, Japan, and the National Institutes of Health, USA.

### *in situ* hybridization (ISH)

Coronal sections were cut at 40 μ m thickness for single-color ISH, 15–20 μ m for double ISH, and 15–20 μ m for tracer-ISH using a freezing microtome. The detailed description of ISH method can be found in a previous paper (Watakabe et al., 2010) or on the web (http://www.nibb.ac.jp/brish/). The FastBlue injection and the subsequent ISH were performed as previously (Watakabe et al., 2007). The FluoroGold injection and the following ISH was performed as previously (Watakabe et al., 2012).

The ISH probes for the mouse and monkey nurr1 and VGluT1 gene have been described previously (Watakabe et al., 2007). To clone cDNA fragments of the cux2, netrinG2, and latexin genes, PCR was performed on the mouse and monkey cDNAs using the following primer sets. For the cux2 gene, 5′-TGGAGTGGGAGTTCTGAAAG-3′ and 5′-GGAAACTTCCTGGGTTGTGC-3′; for the netrinG2 gene, 5′-ATGCCGAAGGCCTCCATGCA-3′, and 5′-CTGTCACAATTTGAGAGTCTGC-3′. For the latexin gene, 5′-TTGGTGGCACAGAACTACATCA-3′ and 5′-GTGACACTTTGGGATTATTTGG-3′.

### Lucifer yellow injection

To investigate dendritic morphology, Lucifer Yellow injection was performed essentially as described previously (Oga et al., 2013). Briefly, 11-week old male BL6 mice (~ 30 g) or 7-week old male Wistar rats (~ 150 g) were perfused with 30 and 150 ml of 4% paraformaldehyde/0.1 M phosphate buffer, at the rate of 1 or 10 ml/min for mice and rats, respectively. The fixed brain was excised and sliced at 200 μ m, in the coronal plane, using a vibratome. After counterstaining with 1 μ g/ml DAPI, Lucifer Yellow was injected into individual cells under the visual guidance of ultraviolet illumination. In the low-power view of the dark field image, the insular/claustral regions appeared as a dark column next to the rhinal fissure, which itself was sandwiched between the brighter neocortex and piriform cortices. The injections were targeted from the posterior side of the slices.

To identify the precise border between the claustrum and the insular cortex, and to enhance the fluorescent signals of the Lucifer Yellow, the injected slices were processed for immunostaining using the biotinylated rabbit antibody to Lucifer Yellow (Invitrogen, A5751) and the mouse monoclonal antibody to latexin (Arimatsu et al., 1992, 1:500) or parvalbumin (Swant235; 1:1000) in 2% bovine serum albumin, 1% Triton X-100, 0.1% sodium azide, and 5% sucrose in 0.1 M phosphate buffer for 5–7 days at room temperature, followed by detection with streptavidin-Alexa488 and Cy3-conjugated anti-mouse antibody.

### Data acquisition and processing

The fluorescent images were captured by an Olympus DP71 digital camera attached to a BX51 microscope (Olympus). The confocal images were taken by a Nikon confocal laser microscope system A1. Maximum intensity projection images for the confocal data were created by NIS-Elements imaging software (Nikon). All the images were processed by Adobe photoshop for proper contrast for presentation. The dendritic morphologies of the Lucifer Yellow injected cells were traced using Imaris FilamentTracer (Bitplain AG, Zurich, Switzerland). To examine the polarity of dendrite extensions, the horizontal and vertical axes were determined, using the low-power image (10×), with the external capsule horizontal below the claustrum and the insula above the claustrum. The tips of the dendrites and the cell body of the targeted cells were marked and their X–Y positions within the image were measured using ImageJ (http://imagej.nih.gov/ij/). The distance between the “Top,” “Bottom,” “Right,” and “Left” borders and the center of the cell body was calculated based on these values.

## Results

### cux2 and netrinG2 mRNAs are expressed in the nurr1^+^ neurons in the mouse cortex and claustrum

To clarify whether the cux2 and netrin G2 genes are expressed in the same cell populations that express latexin and nurr1, we first performed double ISH using these gene markers in the mouse cortex and later in the macaque brain.

**Figure 1:**
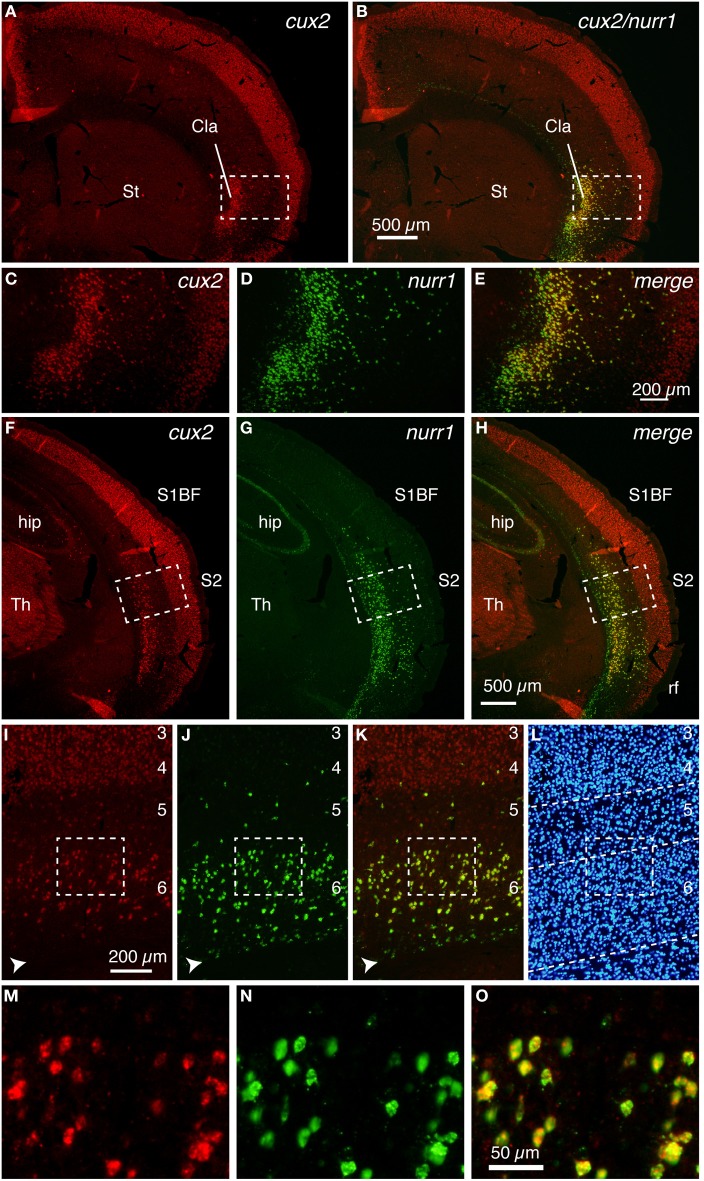
**Colocalization of cux2 and nurr1 mRNAs in the claustrum and cortical deep layer of the mouse brain**. DIG-labeled cux2 antisense probe (detected in red) and FITC-labeled nurr1 antisense probe (detected in green) were hybridized to the coronal sections of the mouse brain. **(A,B)** Double ISH of cux2 (red) and nurr1 (green) mRNAs in the frontal section. **(A)** shows only the red channel (cux2 signal), while **(B)** shows the merged view for red and green channels of the same section. **(C–E)** The dashed rectangles in **(A,B)** were magnified and shown for different channels. **(C,D)** show the signals for cux2 and nurr1 mRNAs and **(E)** shows the merged image of **(C,D)**. **(F–H)** Double ISH of cux2 and nurr1 in the more posterior section. **(F,G)** show red (cux2 signal) and green (nurr1) channels, while **(H)** shows the merged view for red and green channels. **(I–K)** Dashed rectangles in **(F–K)** were magnified. The arrowheads in panels **(I–K)** indicate the nurr1^+^/cux2^−^ cells in layer 6b. **(L)** Hoechst nuclear staining to confirm lamina boundaries in **(I–K)**. The dashed lines indicate the lamina borders determined by the density of nuclear staining. **(M–O)** Dashed rectangles in **(I–K)** were magnified. Note exact colocalization of the two mRNAs in **(O)**. Cla, claustrum; hip, hippocampus; rf, rhinal fissure; S1BF, somatosensory barrel field; S2, secondary somatosensory area; St, striatum; Th, thalamus.

Figure 1A shows the typical upper layer (layers 2–4) expression of cux2 mRNA, together with latexin-like expression in the lateral cortex of mice. By double ISH, we found that the cux2 and nurr1 genes are densely expressed in the claustrum and co-expressed, as expected from such high density of expression (Figure 1B). In the adjacent insular cortex, they exhibited scattered pattern, but still mostly co-expressed in layers 5–6 (Figures 1C–E). The high coincidence of expression even in such a scattered pattern strongly suggests that the two genes are expressed in a cell-type specific fashion. The co-expression was not observed in layer 6b, where we observed nurr1 mRNA-single positive cells. This subpopulation is considered to correspond to the latexin^−^/CTGF^+^ subpopulation of nurr1^+^ cells (Arimatsu et al., 2003; Watakabe et al., 2007). More posteriorly, both cux2 and nurr1 mRNA expression were observed in layer 6 of S2 (Figures 1F–H) and this was precisely co-localized (Figures 1I–O).

**Figure 2:**
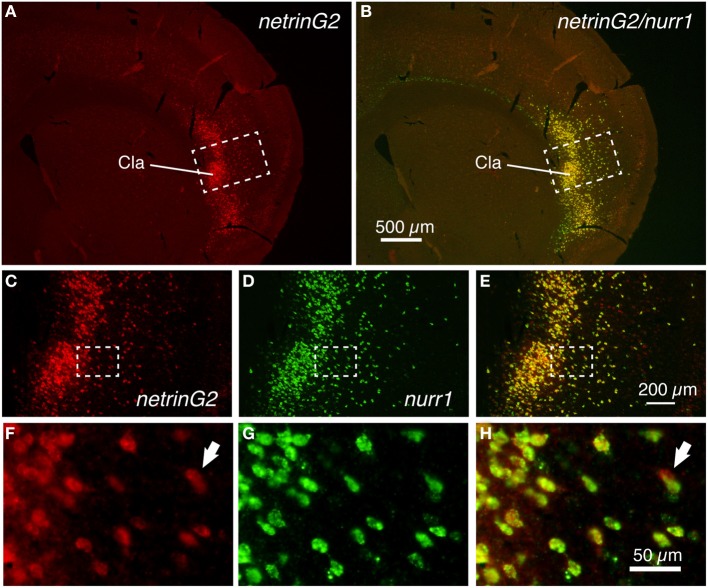
**Colocalization of netrinG2 and nurr1 mRNAs in the mouse brain**. **(A,B)** Double ISH of netrinG2 (red) and nurr1 (green) mRNAs in the mouse frontal section. **(A)** shows only the red channel (netrinG2), while **(B)** shows the merged view for red and green channels of the same section. **(C–E)** Dashed rectangles in **(A,B)** were magnified and shown for netrinG2 **(C)**, nurr1 **(D)**, and merged **(E)** signals. **(F–H)** Dashed rectangles in **(C–E)** were magnified. The arrows in **(F,H)** indicate a netrinG2-single positive cell.

We next examined the expression pattern of the netrinG2 gene in the mouse brain. As Figure 2A shows, the netrinG2 gene exhibited a latexin-like pattern in the adult mouse cortex. Importantly, double ISH demonstrated that netrinG2 and nurr1 mRNAs are exactly co-localized in the claustrum and in the deep layer neurons in the adjacent cortical areas (Figures 2A–H). Like latexin, the netrinG2 gene was not expressed in layer 6b. Other than that, we observed few neurons that expressed netrinG2 mRNA but not nurr1 mRNA (Figures 2F–H, arrows). We conclude that latexin, nurr1, cux2, and netrinG2 gene are all expressed in the same neuronal population that is enriched both in the claustrum and in layer 6 and some in layer 5 of the mouse lateral cortical areas.

### Expression profiling of monkey claustrum and insular cortex for the cux2, netrinG2, latexin, nurr1, and VGluT1 genes

**Figure 3:**
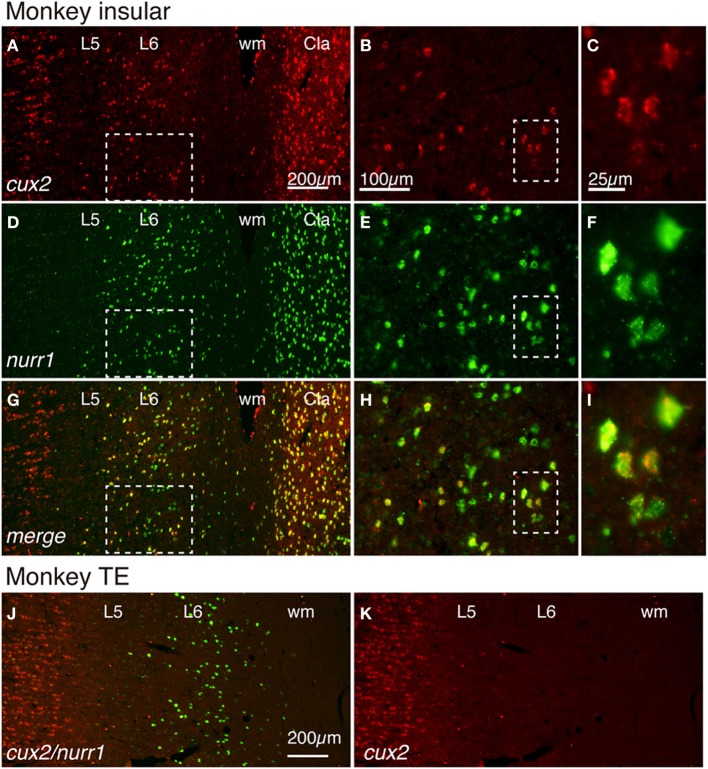
**Colocalization of cux2 and nurr1 mRNAs in the claustrum and insular cortex of the monkey brain**. **(A,D,G)** Double ISH of cux2 (red) and nurr1 (green) mRNAs in the monkey brain section that spans the deep layers 5 (L5) and 6 (L6) of the insular cortex and the claustrum. Left side is to the pia matter. Red **(A)**, green **(D)**, and merged **(G)** channels were shown for the same section to demonstrate the colocalization of cux2 and nurr1 mRNA signals. **(B,E,H)** Dashed rectangles in **(A,D,G)** were magnified. **(C,F,I)** Dashed rectangles in **(B,E,H)** were magnified. **(J,K)** Double ISH of cux2 and nurr1 mRNAs in monkey TE. In **(J)**, both cux2 (red) and nurr1 (green) mRNA signals are shown as merged, while in **(K)**, only the cux2 mRNA signals are shown. Note the absence of cux2 mRNA signals in the deep layers in this area. Cla, claustrum; wm, white matter.

If the cell-type specific expressions of latexin, nurr1, cux2, and netrinG2 genes have functional importance, we might expect their expression patterns to be conserved across species. To test this, we examined the expression of these genes in the monkey claustrum and the adjacent insular cortex. We first examined whether cux2 and nurr1 mRNAs are co-expressed in the monkey cortex. Figure 3 shows double ISH of cux2 and nurr1 mRNAs in the monkey insular cortex and the claustrum. The double ISH demonstrated that cux2 mRNA is co-expressed with nurr1 mRNA in exactly the same cells, both in layer 6 and claustrum (Figures 3A–I); but we observed some nurr1^+^ neurons in the lower half of layer 6 that do not co-express cux2 mRNA (compare Figures 3B,E,H). This cux2^−^/nurr1^+^ subpopulation is considered to correspond to the CTGF^+^/nurr1^+^ population that we previously described in monkeys (Watakabe et al., 2007). In addition, cux2 mRNA was also expressed in the upper layers, which is consistent with the mouse and human data (Nieto et al., 2004; Arion et al., 2007). Intriguingly, unlike nurr1 mRNA, cux2 mRNA was not expressed in layer 6 neurons of all areas. In area TE, for example, cux2 mRNA expression was restricted to the upper layers (Figures 3J,K), suggesting further differentiation of nurr1-mRNA expressing cortical neurons.

**Figure 4:**
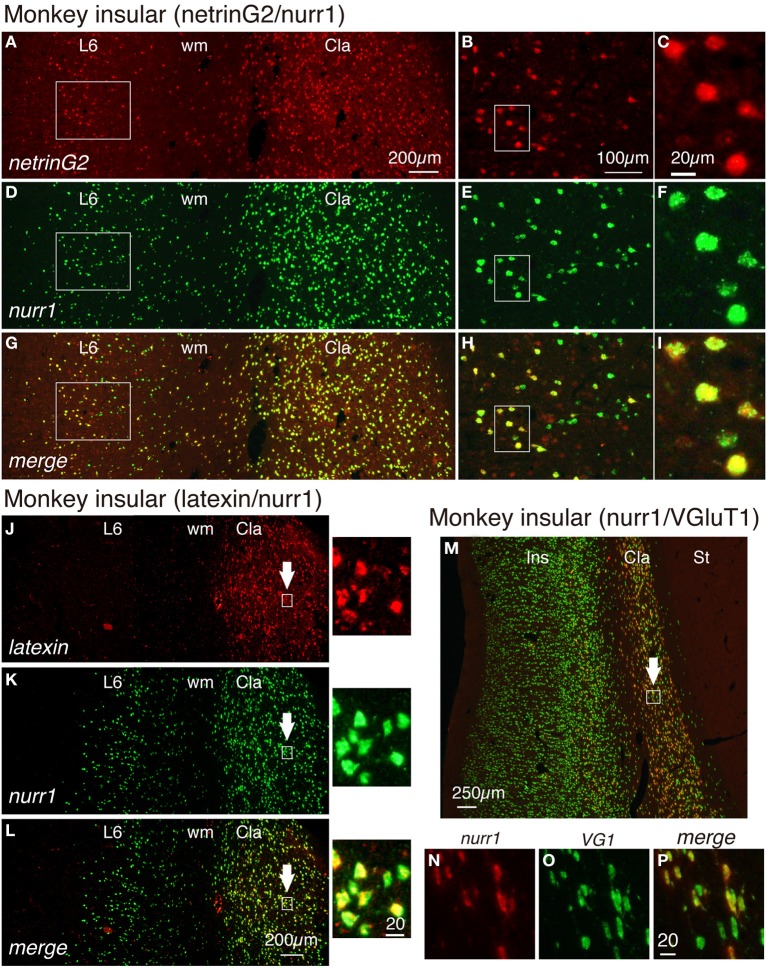
**Gene expression profiling for insular and claustral neurons by netrin G2, nurr1, latexin, and VGluT1 genes**. **(A,D,G)** Double ISH of netrinG2 (red) and nurr1 (green) mRNAs in the monkey brain section that spans layer 6 (L6) of the insular cortex and the claustrum (Cla). Left side is to the pia matter. Red **(A)**, green **(D)**, and merged **(G)** channels were shown for the same section to demonstrate the colocalization of netrinG2 and nurr1 mRNA signals. **(B,E,H)** Rectangles in **(A,D,G)** were magnified. **(C,F,I)** Rectangles in **(B,E,H)** were magnified. **(J–L)** Double ISH of latexin (red) and nurr1 (green) mRNAs in the monkey brain section that spans layer 6 (L6) of the insular cortex and the claustrum (Cla). Left side is to the pia matter. The rectangles in the claustrum indicated by the arrows were magnified on the right side of each panel. **(M)** A merged view for double ISH of nurr1 (red) and VGluT1 (green) mRNAs in the monkey brain section that spans the entire layers of the insular cortex (Ins), the claustrum (Cla) and the striatum (St) is shown. Left side is to the pia matter. Red **(N)**, green **(O)**, and merged **(P)** channels were shown for the rectangle in the claustrum indicated by the white arrow. All the nurr1^+^ neurons were VGluT1^+^ glutamatergic in this analysis, while some VGluT1^+^ cells in the claustrum exhibited only low level expression of nurr1 mRNA (compare **N–P**). Cla, claustrum; Ins, insular cortex; St, striatum; wm, white matter.

In a previous study, the netrinG2 gene was reported to be expressed in the claustrum and layer 6 neurons of the insular cortex (Miyashita et al., 2005). As shown in Figures 4A–H, we found by double ISH that the netrinG2 gene is co-expressed with the nurr1 gene in layer 6 of the insular cortex as well as in the claustrum. Like cux2 gene, we observed nurr1mRNA-positive cells in layer 6b that do not express netrinG2 mRNA. The expression of netrinG2 and nurr1 mRNAs in the insular cortex, however, generally coincided in limited subpopulation. The coexpression of netrinG2 and nurr1 genes were observed in all the areas we examined including, frontal, motor, somatosensory, temporal, and visual areas (data not shown).

We have examined the latexin mRNA expression in the monkey cortex. As shown in Figures 4J–L, latexin mRNA was not expressed in the insular cortex. However, we found it to be expressed in the claustrum where it co-localized with nurr1 mRNA. The expression of latexin mRNA was somewhat heterogeneous and some claustral nurr1 mRNA-positieve cells expressed only low levels of latexin mRNA (see the magnified images indicated by the arrows in Figures 4J–L).

In the adult rodent brain, claustral neurons are reported to express vesicle glutamate transporter 2 (VGluT2) mRNA (Fremeau et al., 2001; Hur and Zaborszky, 2005). However, we detected only low level of VGluT2 mRNA in the monkey claustrum in comparison to that in the thalamus (data not shown). So, we tested the expression of VGluT1 mRNA, which has been used as a marker for excitatory glutamatergic neurons of the cerebral cortex (Komatsu et al., 2005). As shown in Figures 4M–P, VGluT1 mRNA was expressed in the insular cortex and claustrum of the monkey brain. In the claustrum, most but not all of the VGluT1mRNA-positive neurons co-expressed nurr1 mRNA. VGluT1 mRNA-positive/nurr1 mRNA-negative cells were present in the white matter that surround the claustrum (Figure 4M). We conclude that claustral expression of latexin, nurr1, cux2, and netrinG2 genes is conserved across mice and monkeys, whereas cortical expression patterns are modified in monkeys.

### Claustral nurr1 mRNA-positive neurons have cortical projections

**Figure 5:**
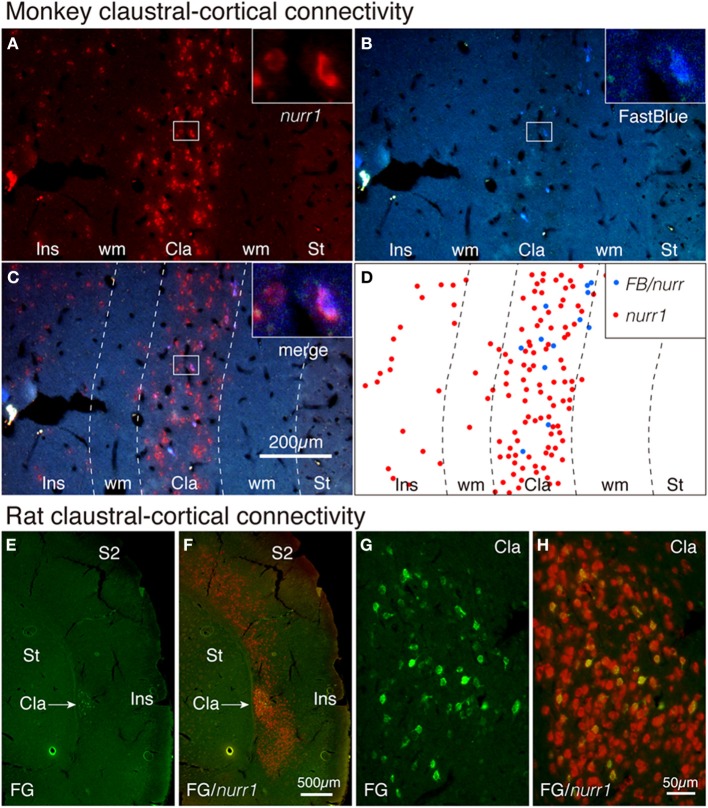
**Tracer-ISH of monkey and rat claustrum. (A–D)** FastBlue was injected into areas V4 and 7a and several claustral cells were retrogradely labeled **(B,D)**. The same section processed for nurr1 ISH **(A,C)** exhibited nurr1 expression in all the FastBlue-labeled cells (see the insets). The rectangles within the claustrum are shown magnified in the insets for each panel. **(D)** indicate the schematic plot for **(C)**; the nurr1 single-positive cells were plotted in red, and the nurr1/FastBlue double positive cells were plotted in blue. **(E,F)** FluoroGold was injected into V1 and the claustral cells were retrogradely labeled. The same section was processed for nurr1 ISH. **(G,H)** The claustral regions of **(A,B)** were magnified. Cla, claustrum; Ins, insular cortex; S2, secondary somatosensory area; ST, striatum; wm, white matter.

It is well established that claustral cells have widespread cortical projections (LeVay and Sherk, 1981; Carey and Neal, 1985; Tanne-Gariepy et al., 2002). To directly test whether such projections originate from the nurr1^+^ populations, we carried out tracer-ISH experiments. FastBlue was injected into areas V4 and 7a of a monkey to produce retrogradely labeled neurons. As shown in Figures 5B–D, all the FastBlue-positive cells expressed nurr1 mRNA in the claustrum. We injected FluoroGold into V1 in a rat and found retrograde labeling in the claustrum (Figure 5E). The FluoroGold-positive cells in the claustrum expressed nurr1 mRNA (Figures 5E–H).

### ISH of nurr1 gene in the embryonic monkey and postnatal mouse

**Figure 6:**
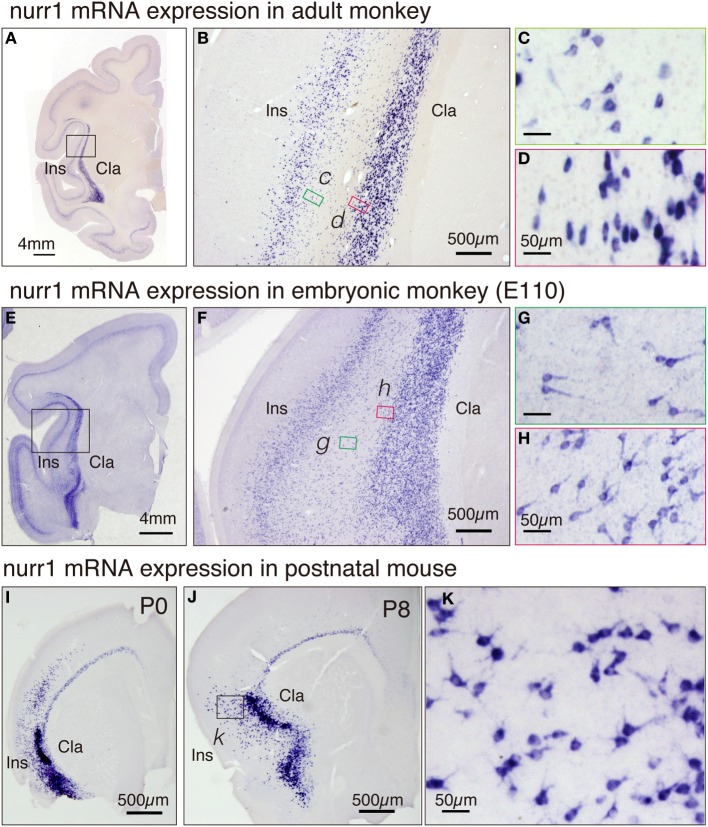
**Expression of nurr1 mRNA in the embryonic monkey and postnatal mice. (A)** Single ISH of nurr1 mRNA in the coronal section of the adult monkey brain hemisphere. Note a dense expression of nurr1 mRNA in the claustrum and layer 6 of the insular cortex. **(B)** The rectangle in **(A)** was magnified. Note the scattered cell population in the white matter between the insular cortex and the claustrum. **(C,D)** The rectangles in **(B)** were magnified. **(C)** shows the shapes of the cell bodies at the bottom of the insular cortex, most of which were typical pyramidal except those at the very bottom (right side of the panel). **(D)** shows the shapes of the cell bodies at the top of the claustrum, which are elongated in horizontal direction. **(E)** ISH of nurr1 mRNA in the coronal section of the embryonic E110 monkey brain hemisphere. **(F)** The rectangle in **(E)** was magnified. Note the abundant scattered cell population in the white matter between the insular cortex and the claustrum. **(G,H)** The rectangles in **(F)** were magnified. **(G)** shows the shapes of the cell bodies in the white matter, many of which appear like inverted pyramidal cells. **(H)** shows the cell bodies at the top of the claustram, which are irregularly shaped. **(I,J)** ISH of nurr1 mRNA in the coronal section of the postnatal P0 and P8 mouse brain hemisphere. **(K)** The rectangle in **(J)** was magnified. Note similarity of the shapes of the cells to those in the monkey claustrum **(G)**.

To categorize cell types in the adult brain, it is informative to investigate the ontogenic origin. Toward this goal, we investigated the expression of nurr1 gene in the embryonic monkey brain. In Figures 6E–H, we illustrate the nurr1 mRNA distribution in the embryonic E110 monkey. We found that the adult pattern of nurr1 mRNA distribution (Figures 6A–D) is already established at this stage. One difference was the abundance of nurr1 mRNA-positive cells in the white matter that separates the insular cortex and the claustrum, as if these were persistent residuals of the migratory cells (Figure 6F). Such a white matter population was also present in the adult monkey brain, although fewer than that in the E110 brain (Figure 6B). The mRNA distribution in the proximal dendrites was well visualized in the embryonic tissue (Figures 6G,H) to show morphology of the cell body. Inverted pyramids (Figure 6H) or multipolar cells (Figure 6G) were easily observed in the claustrum and the white matter, but “standard” pyramidal neurons (i.e., with a discernible apical dendrites oriented toward the pia), only rarely.

For comparison, we investigated the distribution of nurr1 mRNA in P0 and P8 mice (Figures 6I–K). The distribution of nurr1 mRNA was almost identical to that in the adult brain at this stage. As in the embryonic monkey, the proximal dendrites were more evident in the postnatal mice (Figures 6I–K).

### Morphological analyses of claustral neurons in rodents by lucifer yellow injection

**Figure 7:**
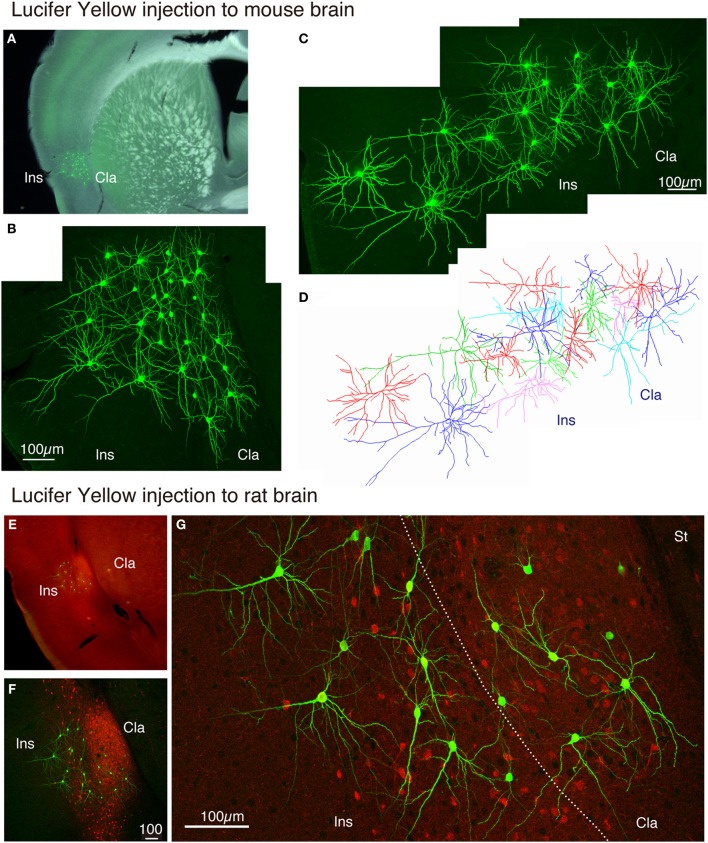
**Lucifer Yellow injection to the mouse and rat claustral neurons. (A)** Dark field image of the mouse slice that received Lucifer Yellow injection to the insular/claustral region. The fluorescent image of the antibody-enhanced Lucifer Yellow-filled neurons is superimposed to indicate the location of clustered injection. **(B)** Maximal projection stacks of confocal sections for magnified view of the insular/claustral region of **(A)**. Note the meshwork of dendrites that cover the insular cortex and the claustrum. **(C)** Another injection into the mouse slice. The pia is to the left. **(D)** Reconstruction of the Lucifer Yellow injected neurons shown in **(C)**. Different cells are shown by different colors for better representation of each cell. **(E)** Merged view for latexin (red) and Lucifer Yellow (green)-stained slice of the rat brain. The densely stained oval at the bottom of the insular cortex is considered as the claustrum. **(F)** Maximal projection stacks of confocal sections for magnified view of the insular/claustral region of **(E)**. Note that the insular/clautral border can be delineated by the dense fiber staining of latexin within the claustrum. Latexin^+^ neurons were observed in a scattered manner outside the claustrum as well. **(G)** Magnified view of the insular/claustral border of **(F)**. Note the differential dendritic arborization of the claustral and insular cells. The dotted line indicates the insular/claustral border identified on the basis of latexin fiber staining. Cla, claustrum; Ins, insular cortex; St, striatum.

The claustrum is separated from the insular cortex by white matter in monkeys, but in the rodent brain, the two structures appear continuous. We wondered whether the neurons in the insular cortex and claustrum of rodents have segregated or intermingled dendrites. To test this, we injected Lucifer Yellow randomly within the mouse insular/claustral regions. As shown in Figures 7A–D, the random injection resulted in a meshwork of dendrites that appeared to cover both insular cortex and claustrum. On closer examination, however, dendrites appeared to be differentiable between claustrum and insular cortex. While the upper layer neurons in the insular cortex exhibited typical pyramidal shape with conspicuous, vertically oriented apical dendrites, the lower part of this region that should correspond to the claustrum had neurons without such dendrites. The dye-injected neurons were mostly spiny, although we encountered some smooth neurons as well. In this mouse experiment, we were not sure of the precise border between the insular cortex and the claustrum. With the rat preparations, we were able to stain the slice with monoclonal latexin antibody (Arimatsu et al., 1992) after the Lucifer Yellow injection, which enabled us to determine the precise border between insular cortex and the claustrum.

**Figure 8:**
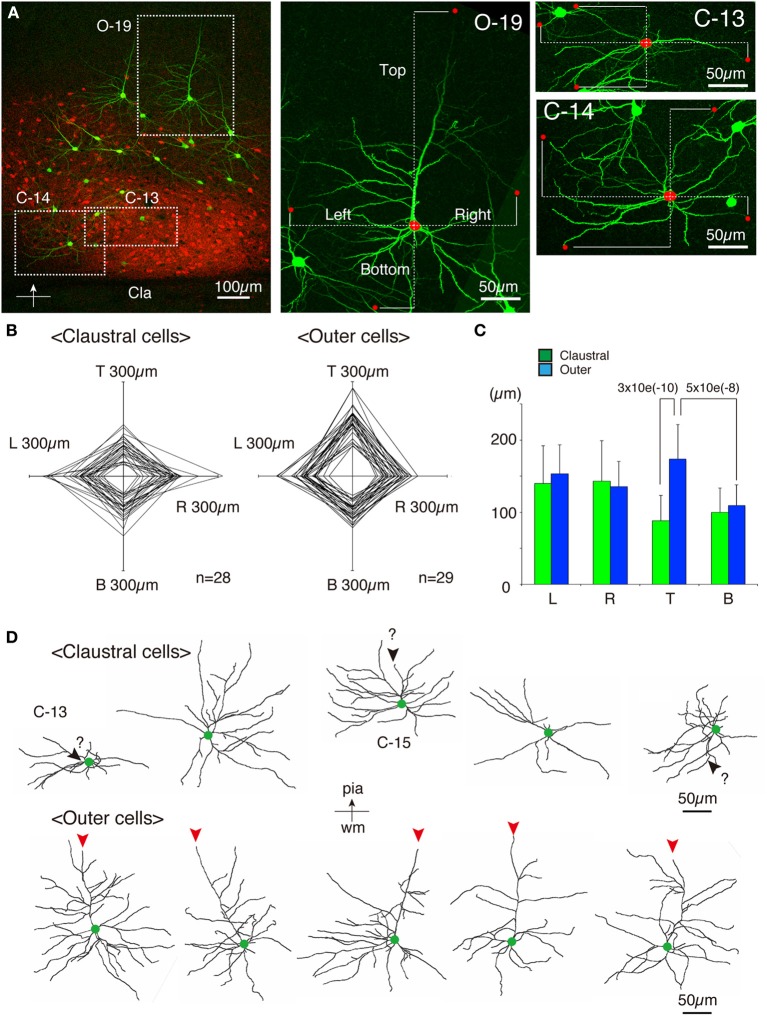
**Quantitative analysis of dendritic field extension for Lucifer-Yellow injected cells. (A)** A typical example of the fluorescent image of the analyzed insular/claustral region. The axis of analysis (shown by the bar and the arrow at the bottom) was determined so that the pia is to the “Top.” This panel was taken from the same section shown in Figures 7E–G. Three analyzed cells, C-13, C-14, and O-19, were magnified on the right. The red dots on each cell indicate the tips of the dendrites that determined the “Top,” “Bottom,” “Right,” and “Left” borders of the dendritic field of the cell of interest. The distance from the center of the cell body to these borders were measured (dotted lines) to examine the polarity of dendritic extensions. Because we wanted to measure the overlaps between the insular and claustral territory, we measured the distance in these max projection images, and not the 3D distance from the tips to the cell body. **(B)** Rader plots for 28 claustral and 29 outer cells chosen from eight slices of two rats were superimposed. The ends of each axis correspond to 300 μ m from the center. The “claustral” cells were determined based on the counterstaining by latexin or parvalubumin. The “outer” cells were those that were located outside the dense latexin-positive regions but within the regions containing scattered latexin^+^ cells. **(C)** The averages and standard deviations for Left (L), Right (R), Top (T), and Bottom (B) values for claustral and outer cells are shown. The difference of the “Top” values between the claustral and outer cells or the difference of the “Top” and “Bottom” values for the outer cells were statistically significant with the indicated *p*-value. **(D)** Five representative examples each of the dendritic reconstruction for the claustral and outer cells by Imaris FilamentTracer. All these neurons are oriented so that the top is to the pia. Green dots indicate the position of the cell bodies. The red arrowheads of the outer cells indicate the apical dendrites that were clearly different from other basal dendrites by morphology. Most of the claustral cells lacked such apical dendrites. However, candidates for apical dendrites which may be oriented toward the anterior or posterior directions (orthogonal to the slice plane) were present in some cells, which were shown by black arrowheads with question marks.

**Figure 9:**
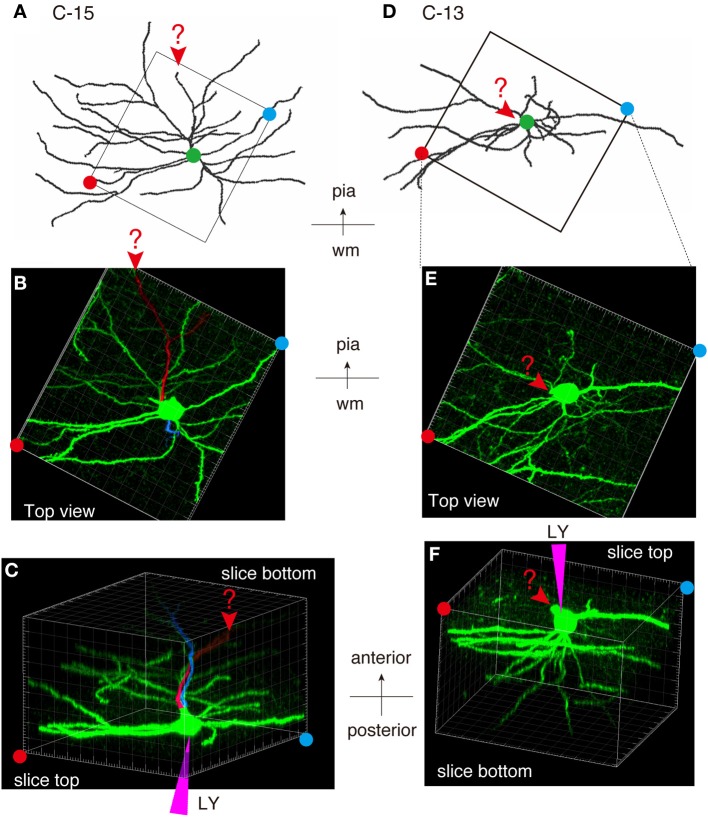
**Examples of atypically oriented pyramidal cells in the claustrum. (A)** Dendritic reconstruction of a claustral cell (C-15) shown in Figure 8D. The region indicated by the square was cut off for 3D reconstruction of the confocal stack images in **(B,C)**. Note the orientation of the neuron: the top is to the pia matter of the insular cortex. **(B)** A maximal projection stack of confocal images of the region shown by a rectangle in **(A)** was viewed from the top surface of the slice. In this view, there does not appear to exist apical dendrites. **(C)** The 3D reconstruction of the confocal images for C-15 was rotated so that the slice is viewed obliquely from the bottom side. Note the positions of the red and blue circles, which indicated the slice tops. The purple triangle represents the pipette position for Lucifer Yellow (LY) injection. The candidate primary and secondary apical dendrites that are oriented toward the bottom of the slice were colored in red and blue, respectively. The arrowheads and the question mark indicates the potential apical dendrites that may be severed. Since we injected from the posterior side of the coronal slice, this means that the candidate apical dendrites are oriented toward the anterior side of the mouse brain. **(D–F)** Dendritic reconstruction of a claustral cell (C-13). The same as for C-15. The rotation of the 3D-reconstruction revealed an appendage that appears like an apical dendrite severed at the slice top surface.

As shown in Figures 7E,F, 8A, the heavy fiber staining of the latexin antibody clearly delimited the border between the insular cortex and the claustrum for the slices used for intracellular Lucifer Yellow injections. Morphologically, the claustral and insular cells were similar to those in the mouse cortex in that the claustral cells lacked apical dendrites and tended to extend dendrites within the claustrum (Figure 7G). To quantify this feature, we measured the extent of dendritic elongation in the coronal plane in maximum intensity projection images as detailed in the methods section. Figure 8B shows the radar charts for 28 claustral cells (e.g., C-13, C-14) and 29 cells outside the claustrum (outer cells; e.g., O-19). Only the spiny neurons were chosen for this analysis. The difference of dendritic elaboration was clearly visible in this panel and the average values were statistically significant (Figure 8C). Figure 8D show five representative tracings for claustral and outer cell dendrites. As these images show, the cell bodies of the outer cells were generally “pyramidal” and had one apical dendrite elongated toward the pial surface (red arrowhead). On the other hand, the claustral cells generally did not have typical apical dendrites that elongate toward the pial surface. In the 3D image, some claustral cells appeared to have “apical” dendrites oriented vertically to the slice surface. Cell C-15, for example, had a thick dendrite with secondary branches that orient toward the bottom of the slices (C-15: Figures 9A–C). Since we always injected from the posterior side of the coronal slice, this means that C-15 may have a dendrite oriented toward the anterior side. Conversely, cell C-13 had a protrusion that looks like a severed dendrite (Figures 9C,D). C-13 may have a dendrite toward the posterior side. These examples suggest that lack of vertically orienting apical dendrites for at least some claustral cells may be because they are oriented along the longitudinal axis. We conclude that while there does not appear to exist clear-cut border of dendritic fields for claustral and insular cells, the dendrites of these cells are mostly contained within each territory.

## Discussion

In this paper, we set out to ask whether the claustral neurons are best considered as belonging to the cortex or the basal ganglia, in part using gene expression as a criterion. As we have demonstrated, nurr1, cux2, and netrinG2 genes are all expressed in both claustrum and cortical layer 6 in rodents and monkeys (summarized in Table 1). This observation supports previous proposals based on molecular marker expression that would favor a cortical phenotype of claustral neurons (Miyashita et al., 2005; Pirone et al., 2012). On the other hand, (1) latexin mRNA was expressed only in the claustrum in macaques and (2) cux2 mRNA was expressed only in the insular cortex and the claustrum (in contrast with the widespread cortical expression of nurr1 and netrinG2 mRNAs). Considering their sparse distribution, these genes would not be co-expressed simply by coincidence in the cortex. Despite obvious species difference, the exact matching of distribution outside claustrum demonstrates the significant similarity of the claustral cells and a subtype of cortical deep layer neuron.

**Table 1:** **Summary of expression patterns for claustral enriched genes**.

	**Gene**	**latexin**	**nurr1**	**cux2**	**netrinG2**	**cux2^+^/nurr1^+^**	**netrinG2^+^/nurr1^+^**
Rodent	Cortex (upper)	No	No	Yes	No	No	No
	Cortex (deep)	Yes^* 1^	Yes^* 1^	Yes^* 1^	Yes^* 1^	Yes^* 1^	Yes^* 1^
	Cortex (layer 6b)	No	Yes	No	No	No	No
	Claustrum	Yes	Yes	Yes	Yes	Yes	Yes
Monkey	Cortex (upper)	No	No	Yes	No	No	No
	Cortex (deep)	No	Yes^* 2^	Yes^* 3^	Yes^* 4^	Only upper layer 6	Only upper layer 6
	Claustrum	Yes	Yes	Yes	Yes	Yes	Yes

*1 , Only in the lateral cortical areas including the insular cortex, S2, and others;

*2 , Both upper and lower part of layer 6 across areas;

*3 , Only in the insular cortex, not inV1, S1, M1, TE, or frontal areas;

*4 , Only in the upper part of layer 6 but across areas including V1, S1, M1, TE, or frontal areas.

The previous literature has identified several candidates for claustral-specific genes; so far these seem to have cortical expression as well. For example, a proteomic analysis has identified G-protein gamma2 (Gng2) subunit as claustral-specific (Mathur et al., 2009). However, Pirone et al., detected Gng2 immunoreactivity in the insular cortex of human brain (Pirone et al., 2012); and Allen brain atlas data (http://mouse.brain-map.org/) show that Gng2 gene is expressed in both the mouse cortex and claustrum in a latexin-like pattern. mRNA for another gene, LGR7, is reported also to exhibit a latexin-like pattern of expression (Piccenna et al., 2005), and we found by double ISH that LGR7 mRNA colocalizes with nurr1 mRNA in the mouse brain (data not shown).

Other more general markers support a cortical, as opposed to striatal signature for claustral neurons. (1) VGluT1 and VGluT2 genes are glutamatergic marker genes, neither of which is expressed in the striatum, where GABAergic cells are the principal cell type. By double ISH, we found that the nurr1^+^ neurons in both mouse and macaque claustrum co-express VGluT1 mRNA (Figure 4 and data not shown). (2) In our previous study, moreover, we reported that excitatory neurons in layer 6 of rats can be classified as either pcp4^+^ or cck^+^ cells, and that nurr1^+^ cells express cck mRNA (Watakabe et al., 2012). In both rodents and monkeys, cck mRNA was expressed in the claustrum but not in the striatum (data not shown). These data are consistent with our tracer-ISH data showing that nurr1^+^ neurons project to the cortex (Figure 5).

In addition, ontogenic data are suggestive of a cortical origin of the claustrum, as has been discussed in previous studies (Miyashita et al., 2005; Pirone et al., 2012). In the embryonic brain, the claustrum expresses TBr1 but not Dlx1, markers; these are associated with pallial (cortical) and striatal structures, respectively (Puelles et al., 2000). Furthermore, autoradiographic birthdating, followed by developmental tracing of the labeled cortical neurons, suggests that claustral neurons are produced in the neocortical neuroepithelium and migrate ventrolaterally to reach their final destination (Bayer and Altman, 1991). Relevant to this point, our ISH data for nurr1 mRNA expression in the embryonic E110 monkey brain revealed many positive cells located within the white matter that separates the insular cortex and the claustrum (Figure 6). The cell body morphology of these neurons is consistent with the migratory pathway proposed by Bayer and Altman (1991) rather than with the typical radial migration for pyramidal cells.

At present, we cannot conclude whether this migratory neuron population is the same subtype as that scattered in layer 6 of the insular and other cortical areas. It is likely that the claustral neurons migrate from distant pallial primordium to the proximity of insular cortex. Note that the radial glia for the insular cortex are reported as anchored at the pial surface and the neuroepithelium of lateral and ventral pallial portions, bypassing the claustrum (Reblet et al., 2002). Further, Mathur and co-workers found that Crym, a marker for cortical deep layer neurons, is expressed in the cells that surround the claustrum (Mathur et al., 2009). Consistent with this finding, we observed VGluT1 mRNA-positive cells in the white matter that surrounds the monkey claustrum.

### Claustral dendrites do not extend into overlying insular cortex

Since Brodmann, there has been discussion that the claustrum may be an additional layer of the insular cortex (reviewed in Swanson and Petrovich, 1998; Swanson, 2000; Edelstein and Denaro, 2004). From this perspective, and given that the claustrum directly abuts the insular cortex in rodents, one might expect some degree of dendritic incursion, at least at the border. Previous Golgi studies (Brand, 1981; LeVay and Sherk, 1981; Braak and Braak, 1982; Mamos, 1984; Mamos et al., 1986; Dinopoulos et al., 1992; Rowniak et al., 1994; Wasilewska and Najdzion, 2001), however, have provided no evidence of dendritic incursion; and, in fact, our Lucifer Yellow filled neurons had dendrites that were clearly skewed, avoiding the overlying insular territory. By simultaneous visualization of the claustral/insular border by latexin immunotaining, we consolidated the past morphological studies. We also performed side-by-side comparison of the insular and claustral cells within the same experimental condition, which clearly showed the different morphological features of these neurons. Our intracellular fills did show some orientation preference of apparently multipolar cells, but the dendritic elongation was along the longitudinal axis (Figure 9). This confirms *in vivo*-labeling and 3D reconstruction demonstrating the existence of a neuron with rostrally-extending apical dendrite in the rostral part of the rat claustrum (Shibuya and Yamamoto, 1998), suggestive of extensive intra-claustral connectivity. Unfortunately, we could not succeed in filling claustral axons in the postmortem slice preparation; but extensive intra-claustral axonal arborization can be inferred from the presence of “hundreds of retrogradely labeled neurons” after localized injections of retrograde tracer in the rat claustrum (Smith and Alloway, 2010).

### The nurr1^+^ subclass of cortical layer 6 neurons

The expression analyses performed in this study strongly suggest the existence of a distinct cortical cell type defined by expression of latexin, nurr1, cux2, and netrinG2 genes in the rodent brain. The expression of these genes in both mouse and monkey claustrum implies similar properties of the claustral and cortical neurons. This could be based in connectivity from the deep cortical layers to claustrum. In our previous study, we proposed that nurr1^+^/CTGF^−^ cells in layer 6 of monkey cortex that cover the entire neocortex may be equivalent to the lateral-restricted nurr1^+^/CTGF-cells in the rodent cortex (Watakabe et al., 2007). Consistent with this idea, we found that netrinG2 mRNA is expressed in the upper subpopulation of nurr1-mRNA positive cells in all the areas examined including motor, somatosensory, TE, frontal and visual cortices (data not shown). Nevertheless, we found that cux2 mRNA is not expressed in the deep layers outside the insular cortex in the macaque cortex. In addition, latexin mRNA was not expressed even in the insular cortex, where cux2 mRNA is co-expressed with nurr1 mRNA. One possible interpretation would be that latexin and cux2 mRNA expression is downregulated as a result of cortical differentiation. Alternatively, the nurr1^+^ neurons in the monkey brain may be ontogenetically independent from those of rodent cortex. Further study is needed to clarify this point and to understand the functional significance of species difference observed here.

### Conflict of interest statement

The authors declare that the research was conducted in the absence of any commercial or financial relationships that could be construed as a potential conflict of interest.
